# Investigating a novel split‐filter dual‐energy CT technique for improving pancreas tumor visibility for radiation therapy

**DOI:** 10.1002/acm2.12435

**Published:** 2018-08-17

**Authors:** Lianna D. Di Maso, Jessie Huang, Michael F. Bassetti, Larry A. DeWerd, Jessica R. Miller

**Affiliations:** ^1^ Department of Medical Physics University of Wisconsin‐Madison Madison WI 53716 USA; ^2^ Department of Human Oncology University of Wisconsin‐Madison Madison WI 53716 USA

**Keywords:** contrast, dual‐energy, pancreas

## Abstract

**Purpose:**

Tumor delineation using conventional CT images can be a challenge for pancreatic adenocarcinoma where contrast between the tumor and surrounding healthy tissue is low. This work investigates the ability of a split‐filter dual‐energy CT (DECT) system to improve pancreatic tumor contrast and contrast‐to‐noise ratio (CNR) for radiation therapy treatment planning.

**Materials and methods:**

Multiphasic scans of 20 pancreatic tumors were acquired using a split‐filter DECT technique with iodinated contrast medium, OMNIPAQUE ^TM^. Analysis was performed on the pancreatic and portal venous phases for several types of DECT images. Pancreatic gross target volume (GTV) contrast and CNR were calculated and analyzed from mixed 120 kVp‐equivalent images and virtual monoenergetic images (VMI) at 57 and 40 keV. The role of iterative reconstruction on DECT images was also investigated. Paired *t*‐tests were used to assess the difference in GTV contrast and CNR among the different images.

**Results:**

The VMIs at 40 keV had a 110% greater image noise compared to the mixed 120 kVp‐equivalent images (*P *<* *0.0001). VMIs at 40 keV increased GTV contrast from 15.9 ± 19.9 HU to 93.7 ± 49.6 HU and CNR from 1.37 ± 2.05 to 3.86 ± 2.78 in comparison to the mixed 120 kVp‐equivalent images. The iterative reconstruction algorithm investigated decreased noise in the VMIs by about 20% and improved CNR by about 30%.

**Conclusions:**

Pancreatic tumor contrast and CNR were significantly improved using VMIs reconstructed from the split‐filter DECT technique, and the use of iterative reconstruction further improved CNR. This gain in tumor contrast may lead to more accurate tumor delineation for radiation therapy treatment planning.

## 1 INTRODUCTION

Pancreatic adenocarcinoma is the fourth‐leading cause of cancer death in the United States.^8^, ^18^ Although surgery is the only established curative treatment option, 80% of patients with pancreatic cancer are not surgical candidates. Radiation therapy offers a local treatment option with recent evidence suggesting that accurately focused dose‐escalated radiation therapy may increase median survival and potential for surgical resection.^3^, ^11^ However, radiation therapy for pancreatic adenocarcinomas can be a challenge because they have poor innate contrast compared to surrounding healthy pancreatic tissue.^12^, ^13^, ^15^ For radiation therapy treatment planning, the lack of tumor contrast makes it difficult to confidently delineate the target with conventional single‐energy computed tomography (SECT) even with iodine contrast.^20^ Accurate tumor delineation is crucial for successful radiation therapy,^14^ particularly in the pancreas where other radiation‐sensitive organs are in close proximity. Recent work has shown that dose escalation can increase survival for pancreatic patients, further increasing the need to clearly visualize and accurately delineate the tumor for treatment planning.^7^, ^17^ Fortunately, recent efforts have been dedicated to using dual‐energy computed tomography (DECT) as an optimal CT modality to increase the detectability of pancreatic tumors.^4^, ^6^, ^8^, ^9^, ^10^, ^12^, ^16^

DECT is an imaging modality that utilizes two different photon spectra to image patient anatomy. Since DECT provides information about the attenuation properties of tissues at two different energies, tissues with similar density but different elemental composition can be differentiated.^2^ DECT images have significant advantages over conventional SECT specifically when imaging the abdomen; DECT applications in the abdomen include, but are not limited to, depicting small liver lesions, differentiating renal masses, and improving depiction of pancreas tumors.^1^ Several studies have been published that investigate the use of DECT techniques for improving pancreas tumor contrast. In these studies, DECT offered improvements in tumor conspicuity and delineation compared to conventional 120 kVp CT.^1^, ^4^, ^8^, ^10^, ^16^

A novel technique for single‐source DECT was recently introduced as an additive feature to the Siemens SOMATOM Definition Edge CT scanner (Siemens Healthcare, Forchheim, Germany). The SOMATOM Definition Edge is now available with a removable gold and tin split‐filter for DECT acquisition, known as TwinBeam (TwinBeam Dual Energy; Siemens). This system may offer a cost‐effective solution for DECT applications in radiation therapy. The TwinBeam system is an innovative DECT modality that utilizes a split‐filter to spatially separate a helical 120 kVp x‐ray beam into a low‐ and high‐energy beam along the longitudinal axis. TwinBeam allows for the low‐ and high‐energy data of the same location in the patient to be acquired within two tube rotations. The temporal coherence between the low‐ and high‐energy acquisition gives TwinBeam the capability to image dynamic contrast, making this modality a candidate for DECT imaging of pancreatic adenocarcinoma where iodine contrast is needed to differentiate healthy pancreatic tissue from tumor. However, in comparison to other dual‐energy techniques that utilize a low‐energy 80 kVp beam and high‐energy 140 kVp beam, the split‐filter technique of TwinBeam has inferior spectral separation.^2^, ^5^ The effects of this limited spectral separation on image quality, specifically in the pancreas have yet to be investigated.

Although some studies have investigated the image quality of TwinBeam DECT scans,^2^, ^5^ none have investigated its utility for radiation therapy treatment planning, nor have any studies investigated the use of TwinBeam for identifying and delineating pancreatic tumors. This work investigates tumor contrast while considering the noise characteristics by calculating tumor contrast‐to‐noise ratios (CNR). CNR offers a more comprehensive view of image quality as both contrast and noise play a role in tumor segmentation during the treatment planning process. To the author's knowledge, there has not been any study to date that investigates the contrast between healthy pancreatic parenchyma and the entire gross target volume (GTV), rather a selected subsection of the GTV through a small region of interest (ROI) within the tumor. The goal of this work is to quantitatively determine if the split‐filter DECT technique of TwinBeam can improve the contrast and the CNR of pancreatic GTVs with the long‐term goal of improving tumor delineation for radiation therapy treatment.

## 2 MATERIALS AND METHODS

### 2.A Patients and CT simulation

Following Institutional Review Board approval, a retrospective study was performed for patients who were treated for pancreatic adenocarcinoma at our institution using radiation therapy between June 2016 and November 2017. The study population included 20 patients (13 male, 7 female) with histologically proven pancreatic adenocarcinoma (mean age 69.6 yr: range: 50–86, mean weight 90.3 kg: range 50.8–146.1). Biopsy results were determined surgically or with fine needle aspiration. The study population included stage IB‐IV pancreatic adenocarcinomas that were resectable, borderline resectable, or unresectable. Two tumors were located in the tail of the pancreas and 18 tumors were located in the head of the pancreas with the longest dimension ranging from about 1.5–4 cm. All patients were simulated on the Siemens SOMATOM Definition Edge CT scanner (Siemens Healthcare, Forchheim, Germany) for radiation therapy planning purposes. Patients were imaged during maximum inhalation breath hold guided using the Varian RMP^TM^ system (Real‐time Position Management, Varian Medical Systems, Palo Alto, CA). To minimize motion, Vac‐Lok^TM^ (CIVCO Radiotherapy) cushions were used as immobilization devices. Each patient received IV nonionized iodine contrast medium, OMNIPAQUE^TM^, during CT simulation and two phases of contrast were imaged. All patients had both the pancreatic and portal venous phase scans, except for one patient who only received a portal venous phase scan. The delays were customized on a patient per patient basis using a bolus tracking technique. The average delay was 32 s (range of 30.5–40 s) and 62 s (range of 54–70 s) for the start of the pancreatic and portal venous phase scans, respectively. The average scan time was 10 s with the pancreas tumor located at the center of the scan. Therefore, the center of the tumor was imaged roughly 37 and 67 s after iodine contrast injection for the pancreatic and portal venous phase scans, respectively.

Due to the added beam filtration of the TwinBeam system, roughly two‐thirds of the x‐ray beam is filtered prior to reaching the patient. As a result, large tube currents are required to achieve CTDI_vol_ similar to conventional SECT acquisitions. Due to tube current limitations, patient size, and scan length requirements for individual patients, the imaging protocol varied from patient to patient. The machine effective mAs ranged between 1350 and 1500 mAs. The automatic tube current modulation was not used, and the CTDI_vol_ ranged from 21.6 to 33.6 mGy. Images were acquired with a pitch of 0.3 to 0.45, a rotation time of either 0.5 or 1 s per rotation and reconstructed at a slice thickness of 3 mm.

### 2.B Image reconstruction

The Siemens *syngo.via* software was used to reconstruct virtual monoenergetic images, called Monoenergetic Plus images, as well as images that mimic the appearance of conventional single‐energy 120 kVp images, called mixed images. A mixed image is a weighted sum with of the low‐ and high‐energy datasets to create an image with HU values equivalent to a SECT image at 120 kVp and is therefore referred to as a 120 kVp‐equivalent image. On the other hand, the virtual monoenergetic images (VMIs) used in this study depict how an object would appear if it was imaged using a monoenergetic x‐ray source and are reconstructed using a novel monoenergetic algorithm (nMERA).^6^ The possible reconstructed energies for a VMI range from 40 to 190 keV. For this study, VMIs were reconstructed at energies from 40 to 90 keV at 5 keV increments to investigate the change in contrast and CNR as a function of energy. Based on this preliminary analysis, the remainder of our study focused on two VMI energies: 40 and 57 keV. The VMI at 40 keV was chosen because it demonstrated the greatest CNR for pancreatic tumors, and the VMI at 57 keV was chosen based on physician initial preference.

Due to the increase in noise for low‐energy VMIs, the role of iterative reconstruction on DECT images was investigated. In addition to filtered back projection (FBP) with the D30 reconstruction kernel, the latest generation of Siemens iterative reconstruction called Advanced Modeled Iterative Reconstruction (ADMIRE; Siemens Healthcare, Forchheim, Germany) using the Q30 reconstruction kernel was also used. ADMIRE is a model‐based iterative reconstruction algorithm designed to decrease noise as well as metal and cone beam image artifacts by analyzing the data in both the Fourier and image domain.^6^, ^19^ ADMIRE was applied to the low‐ and high‐energy datasets individually at a strength of 2 (ADMIRE 2) out of a maximum strength of 5; level 2 represents a low to medium level of noise suppression due to iterative reconstruction.

**Figure 1:**
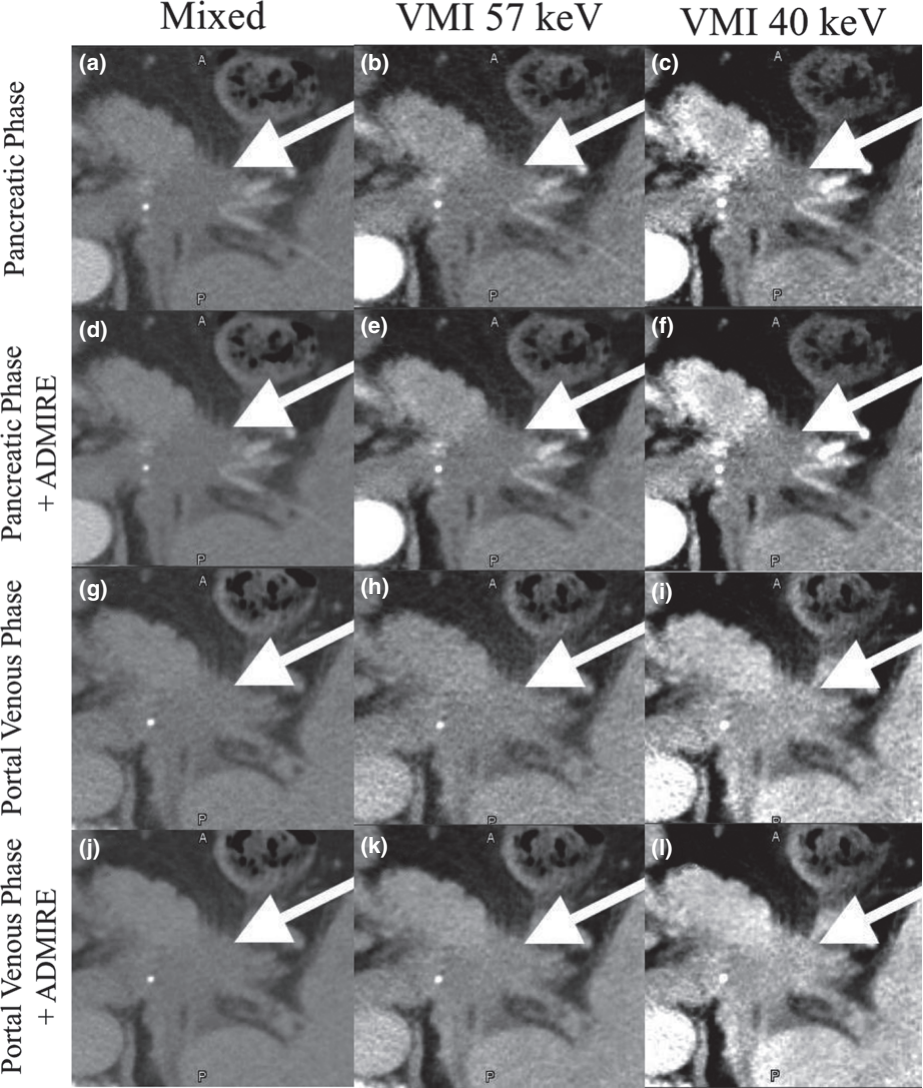
Images from the pancreatic (a–f) and portal venous phase (g–l) with FBP (a–e and g–i) and ADMIRE 2 (d–f and j–l). The arrow indicates the location of the GTV.

In summary, two raw datasets were acquired for each patient: a pancreatic contrast phase and a portal venous contrast phase. For each contrast phase, the raw data was reconstructed using two reconstruction methods, FBP and ADMIRE 2. For each reconstruction method, three dual‐energy images were generated: a mixed 120 kVp‐equivalent image, a VMI at 57 keV image, and a VMI at 40 keV image (Fig. 1).

### 2.C Contrast and contrast‐to‐noise ratio analysis

**Figure 2:**
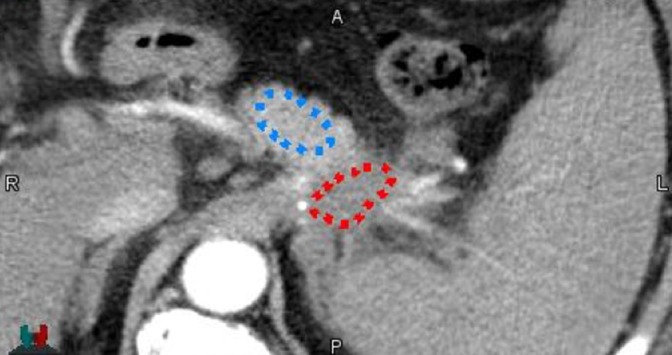
VMI at 57 keV with the pancreatic adenocarcinoma GTV contour in red and the normal pancreas tissue contour in blue.

All dual‐energy images were evaluated using the MIMvista software (MIM Software Inc. Cleveland, OH). Three ROIs were created to evaluate tumor contrast and CNR. This study assessed the whole GTV, as defined by an experienced radiation oncologist on the pancreatic phase VMIs at 57 keV. The attenuation of healthy pancreatic parenchyma was measured using an ROI placed near the GTV within the pancreas that avoided stents, macroscopic vessels, and the pancreatic duct. The placement of the GTV and pancreatic parenchyma ROI contours for a single patient is shown in Fig. 2. The mean and standard deviation of the CT numbers in the GTV and the ROI in the healthy pancreatic parenchyma were calculated for each image dataset. Image noise was assessed with the standard deviation of a ROI located in the erector spinae muscle to avoid the impact of tumor heterogeneity on image noise. The size of this ROI was consistent at 10 mm^2^ among all patients.

The GTV contrast was calculated for each image dataset as ${\text{GTV}}_{\mathrm{contrast}}={\bar{\text{HU}}}_{\mathrm{pancreas}}-{\bar{\text{HU}}}_{\mathrm{GTV}}$, where ${\bar{\text{HU}}}_{\mathrm{pancreas}}$ is the mean CT number of the ROI in healthy pancreatic parenchyma and ${\bar{\text{HU}}}_{\mathrm{GTV}}$, is the mean CT number of the GTV. GTV CNR was also calculated for each image dataset as ${\text{GTV}}_{\mathrm{CNR}}=\frac{{\bar{\text{HU}}}_{\mathrm{pancreas}}-{\bar{\text{HU}}}_{\mathrm{GTV}}}{\sigma }$, where *σ* is the standard deviation of CT numbers of the ROI located in the erector spinae muscle. Contrast and CNR improvement provided by VMIs in comparison to mixed 120 kVp‐equivalent images was calculated.

Differences in contrast and CNR among all reconstructed datasets for a single dual‐energy acquisition were statistically analyzed using analysis of variances (ANOVA). Statistical differences in contrast and CNR between the mixed 120 kVp‐equivalent images and the VMIs reconstructed at 57 and 40 keV were analyzed using paired *t*‐tests. A *P*‐value<0.05 was determined as statistically significant. This study analyzed a total of 39 dual‐energy acquisitions, acquired from 20 patients.

## 3 RESULTS

**Figure 3:**
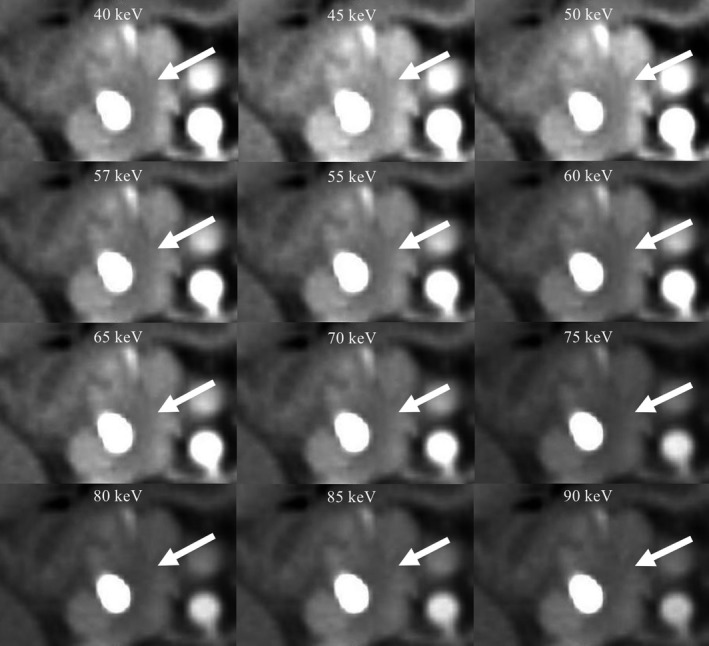
VMIs reconstructed at 40–90 keV created from the FBP pancreatic phase data. The arrow indicates the location of the GTV.

**Figure 4:**
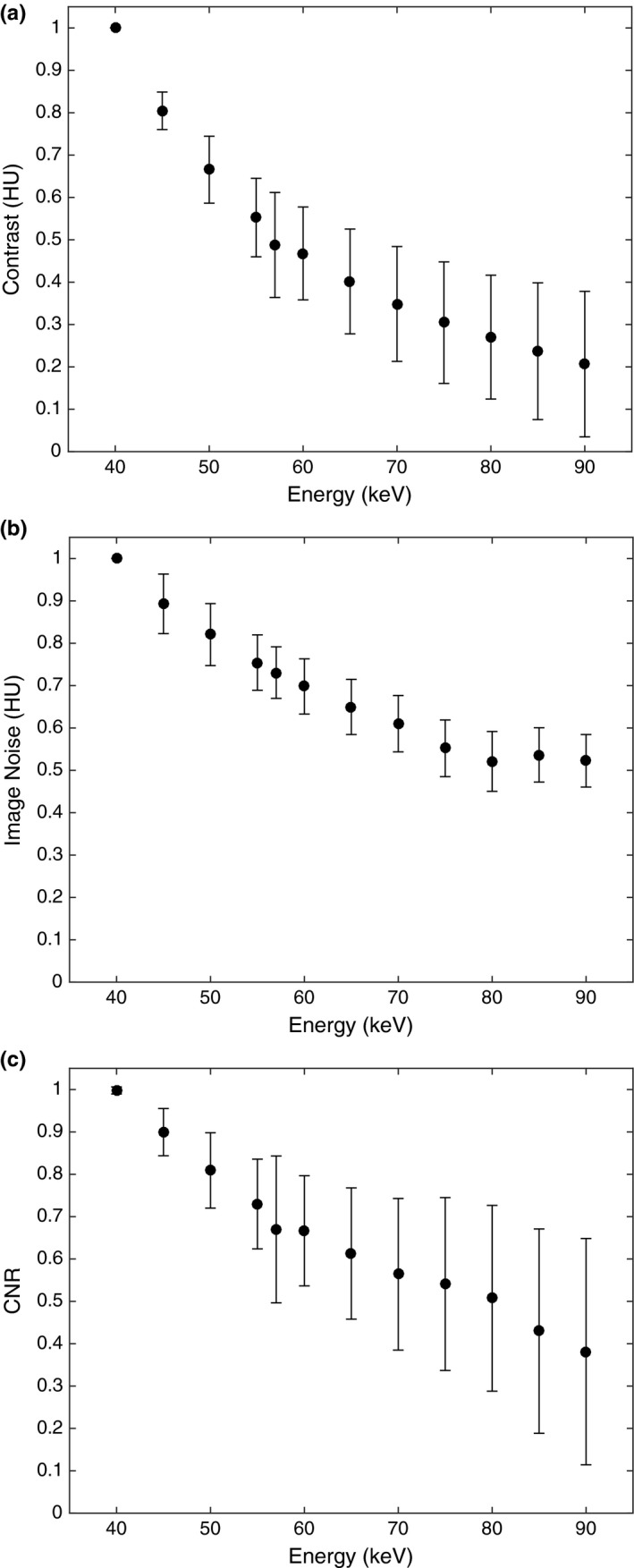
The GTV contrast (a), image noise (b), and CNR (c) for VMIs at energies ranging from 40 to 90 keV normalized to the values at 40 keV. These VMIs were reconstructed from FBP pancreatic phase data. Error bars represent the standard deviation among all patients.

To determine the reconstruction energy for the VMIs that produced the greatest GTV contrast and CNR, VMIs were reconstructed at energies ranging from 40 to 90 keV at 5 keV increments (Fig. 3). Among all patients, the reconstruction energy at 40 keV produced the greatest contrast and CNR. Figure 4 shows the GTV contrast, noise and CNR from VMIs at energies ranging from 40 to 90 keV reconstructed from FBP pancreatic phase data. The remainder of our results focus on comparing VMIs at 40 and 57 keV against mixed 120 kVp‐equivalent images, which were used to represent conventional SECT images.

### 3.A Contrast

**Figure 5:**
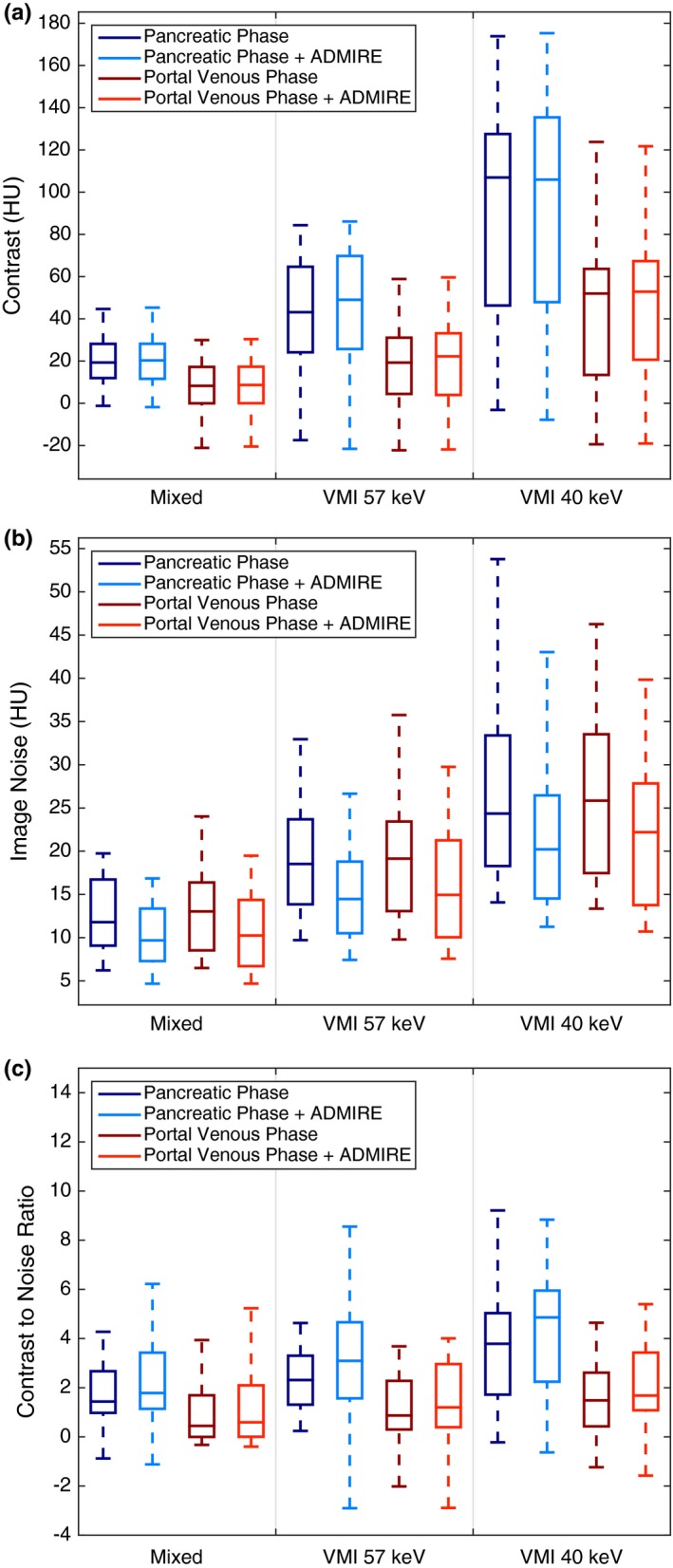
Boxplots of GTV contrast (a), image noise (b), and CNR (c) from both contrast phases with FBP or ADMIRE 2 reconstructed into mixed 120 kVp‐equivalent images (Mixed), virtual monoenergetic images at 57 keV (VMI 57 keV), and at 40 keV (VMI 40 keV).

The mean contrast values for the mixed 120 kVp‐equivalent images, VMIs at 57 keV, and VMIs at 40 keV for both contrast phases are shown in Fig. 5(a). The mean ± standard deviation (SD) GTV contrast for the pancreatic phase datasets using FBP was 15.9 ± 19.9 HU for the mixed 120 kVp‐equivalent images. The VMIs at 57 keV increased the GTV contrast to 40.7 ± 27.7 HU, which represents a 219% increase in contrast (*P *=* *0.0025). The VMIs at 40 keV increased the GTV contrast to 93.7 ± 49.6 HU for a mean contrast improvement of 665% compared to the mixed 120 kVp‐equivalent images (*P *<* *0.0001). The mean ± SD GTV contrast for the portal venous phase datasets using FBP was 6.01 ± 15.2 HU, 16.4 ± 20.9 HU, and 41.5 ± 34.9 HU for the mixed 120 kVp‐equivalent images, VMIs at 57 keV, and the VMIs at 40 keV, respectively (*P *<* *0.0001). The GTV contrast was greater for all pancreatic phase images when compared to the portal venous phase images (*P *<* *0.0001). On average, images reconstructed with ADMIRE had slightly greater contrast but this improvement was statistically insignificant (*P *=* *0.8717). The mean ± SD GTV contrast for the pancreatic and portal venous phase datasets reconstructed with the FBP or ADMIRE and *P*‐values are displayed in Table 1.

**Table 1:** GTV contrast, image noise, and CNR from both contrast phases with FBP or ADMIRE 2 reconstructed into mixed 120 kVp‐equivalent images (Mixed), virtual monoenergetic images at 57 keV (VMI 57 keV), and at 40 keV (VMI 40 keV)

	Mixed	VMI	VMI	*P*‐value^a^	*P*‐value^b^	*P*‐value^c^
		57 keV	40 keV			
Contrast (HU)						
Pancreatic phase	15.9 ± 19.9	40.7 ± 27.7	93.7 ± 49.6	2.50E‐03	1.12E‐07	1.72E‐04
Pancreatic phase + ADMIRE	15.8 ± 19.9	43.8 ± 28.2	96.5 ± 50.4	1.05E‐03	6.94E‐08	1.99E‐04
Portal venous phase	6.01 ± 15.2	16.4 ± 20.9	41.5 ± 34.9	0.0887	2.53E‐04	1.08E‐02
Portal venous phase + ADMIRE	6.23 ± 15.2	19.3 ± 19.7	46.7 ± 31.9	0.0281	1.54E‐05	2.95E‐03
Image noise (HU)						
Pancreatic phase	13.2 ± 4.38	20.3 ± 8.45	28.0 ± 12.5	1.90E‐03	1.35E‐05	0.0278
Pancreatic phase + ADMIRE	10.6 ± 3.69	15.8 ± 6.09	22.2 ± 9.02	2.07E‐03	4.78E‐06	0.0130
Portal venous phase	13.4 ± 5.17	20.6 ± 8.90	27.7 ± 10.6	4.40E‐03	6.50E‐06	0.0315
Portal venous phase + ADMIRE	10.9 ± 4.43	16.5 ± 7.03	23.5 ± 9.37	5.68E‐03	7.81E‐06	0.0155
CNR						
Pancreatic phase	1.37 ± 2.05	2.41 ± 2.15	3.86 ± 2.78	0.140	2.61E‐03	0.0671
Pancreatic phase + ADMIRE	1.69 ± 2.63	3.16 ± 2.83	4.94 ± 3.61	0.105	2.37E‐03	0.0856
Portal venous phase	0.674 ± 1.65	1.15 ± 1.80	1.85 ± 2.30	0.402	0.0750	0.292
Portal venous phase + ADMIRE	0.822 ± 2.11	1.52 ± 2.22	2.42 ± 2.72	0.326	0.0479	0.262

All values are given in mean ± SD except for *P*‐values.

a Paired *t*‐test comparing columns Mixed and VMI 57 keV.

b Paired *t*‐test comparing columns Mixed and VMI 40 keV.

c Paired *t*‐test comparing columns VMI 57 keV and VMI 40 keV.

### 3.B Noise

The mean ± SD image noise of the mixed 120 kVp‐equivalent images, VMIs at 57 keV, and VMIs at 40 keV with the FBP was 13.2 ± 4.38 HU, 20.3 ± 8.45 HU, and 28.0 ± 12.5 HU, respectively, averaged over the pancreatic and portal venous phase datasets (*P *<* *0.0001). There was no difference in image noise between the two contrast phases (*P *=* *0.919). The image noise was 52% higher for the VMIs at 57 keV and 110% higher for VMIs at 40 keV compared to the mixed 120 kVp‐equivalent images. ADMIRE 2 decreased image noise to 10.6 ± 3.69 HU, 15.8 ± 6.09 HU, and 22.2 ± 9.02 HU for the mixed 120 kVp‐equivalent images, VMIs at 57 keV, and VMIs at 40 keV, respectively (*P *<* *0.0001). ADMIRE 2 decreased image noise by 19.3 ± 4.87% throughout all images.

### 3.C Contrast‐to‐noise ratio

The mean CNR for the mixed 120 kVp‐equivalent images and the VMIs reconstructed at 57 and 40 keV for both the pancreatic and portal venous phases are shown in Fig. 5(c). The mean ± SD for GTV CNR for the pancreatic phase datasets reconstructed with FBP was 1.37 ± 2.05, 2.41 ± 2.15, and 3.86 ± 2.78 for the mixed 120 kVp‐equivalent images, VMIs at 57 keV and VMIs at 40 keV, respectively (*P *=* *0.0057). The pancreatic phase VMIs with the FBP at 57 and 40 keV increased CNR by a mean of 109% and 270%, respectively, compared to the mixed 120 kVp‐equivalent images (*P *=* *0.140, *P *=* *0.00261). ADMIRE further improved CNR for all images. For the pancreatic phase, ADMIRE 2 increased CNR in the VMIs at 40 keV from 3.86 ± 2.78 to 4.94 ± 3.61 (*P *<* *0.0001).

## 4 DISCUSSION

In this study, TwinBeam was investigated to improve the delineation of pancreatic adenocarcinoma for radiation therapy treatment planning. VMIs acquired using TwinBeam were compared against mixed 120 kVp‐equivalent images, which served as a baseline since these images represent conventional single‐energy CT images. Entire pancreas GTVs were analyzed rather than small ROIs placed within the tumors because the contrast and CNR calculated from a ROI do not represent the detectability of the entire tumor volume. Significantly greater GTV contrast and CNR was achieved in the low‐energy VMIs, with the greatest CNR occurring at the lowest reconstructed energy of 40 keV. CNR improvements of up to 500% were found from the VMIs at 40 keV when compared to the mixed 120 kVp‐equivalent images.

As expected, the noise of the VMIs increased with a decrease in energy and was the greatest for the VMIs at 40 keV. The use of iterative reconstruction (ADMIRE) at a strength of 2 decreased noise by about 20% throughout all images and therefore further improved the CNR of the VMIs. This is consistent with other published data which demonstrated that low‐contrast detectability is increased by decreasing noise of DECT images using iterative reconstruction.^6^, ^13^, ^19^ These data suggests that the best tumor visibility can be achieved by contouring on VMIs at 40 keV with ADMIRE, although this may still depend on physician preference, window and leveling. While increasing the strength of iterative reconstruction may further improve CNR,^19^ further investigation into the effects of ADMIRE on edge detection and therefore tumor delineation is needed. In addition, further investigation is needed to determine the impact of increased CNR in VMIs on the accuracy of tumor delineation.

The DECT images were acquired at two different contrast phases, pancreatic and portal venous. The pancreatic phase demonstrated greater GTV contrast and CNR compared to the datasets acquired during the portal venous phase, suggesting that the pancreatic phase is superior for tumor delineation. This agrees with published data^4^, ^12^ and is expected because the timing of the pancreatic phase is designed to maximize tumor‐to‐parenchymal attenuation differences. DECT further improved the contrast and CNR for both contrast phases; however, the improvement for both metrics was greater for the pancreatic phase. The mean increase in CNR for the VMIs at 40 keV compared to the mixed 120 kVp‐equivalent images was 8% greater for the pancreatic phase than the portal venous phase. This demonstrated that the TwinBeam system can exploit subtle differences in iodine uptake better than conventional single‐energy imaging.

While the use of TwinBeam DECT for imaging pancreatic adenocarcinomas has not been previously investigated, others have reported improvements in pancreatic tumor contrast and CNR using a fast‐kVp switching and dual‐source DECT.^4^, ^6^, ^16^ Patel et al. calculated contrast and CNR values from VMIs using a fast‐kVp switching DECT. The contrast values from Patel et al. were higher than the contrast values calculated with TwinBeam for comparable VMIs. This discrepancy in contrast is likely attributed to differences in calculation techniques. Patel et al. calculated contrast values using a small ROI optimally placed inside the tumor, while our study calculated contrast using the entire GTV. Incorporating the entire GTV resulted in lower contrast values, however, these values are more relevant for radiation therapy, where the entire tumor volume must be accurately segmented. Also, the image noise from TwinBeam (27.9 ± 11.5 HU for VMIs at 40 keV) was lower than the values reported by Patel et al. (58.9 ± 16.7 HU for the VMIs at 45 keV). The reconstruction algorithm used to create the VMIs from TwinBeam data is a novel monoenergetic reconstruction algorithm (nMERA) that performs regional spatial frequency‐based recombination of high attenuation at low photon energy images and lower image noise at higher photon energies to obtain the best possible image noise level. This algorithm is different than the standard monoenergetic reconstruction algorithm (sMERA).^6^ The difference in noise characteristics between the DECT systems results in different VMI energies producing the greatest CNR. The VMI energy that produced the greatest CNR was 40 keV for the TwinBeam system, while the optimal energy varied by patient (52.5 ± 7.7 keV) for the fast‐kVp switching technique.^16^ Overall, comparable values of CNR were found between TwinBeam and fast‐kVp switching DECT techniques, especially at the optimal energy for each system (3.86 ± 2.78 for TwinBeam and 3.09 ± 2.0 for kVp fast switching).

Frellesen et al. also investigated contrast and CNR values from VMIs for pancreatic tumors but for a dual‐source DECT technique. Frellesen et al. also used a small ROI placed within the pancreatic adenocarcinoma, rather than the entire GTV as used in this study. In comparison to their work, the image noise of mixed 120 kVp‐equivalent images from TwinBeam with ADMIRE 2 (10.7 ± 4.02 HU) are comparable to image noise values from dual‐source DECT (10.69 ± 3.57 HU).^6^ TwinBeam demonstrated inferior CNR values for pancreatic adenocarcinomas when compared to the dual‐source technique for VMIs at 40 keV calculated with nMERA (4.94 ± 3.61 for TwinBeam and 26.29 ± 16.83 for dual‐source). This discrepancy in CNR may partially be attributed to the difference in calculating contrast between using a small ROI and the entire GTV.

This comparison is significant as it quantifies CNR values that are achievable with the limited spectral separation inherent in the TwinBeam system when compared to DECT systems with greater spectral separation. This work also demonstrated comparable image noise of mixed 120 kVp‐equivalent images between TwinBeam and dual‐source systems. This comparison highlights that in addition to the spectral separation between different dual‐energy CT systems, the gain in CNR also depends on the algorithm used to generate monoenergetic images.

TwinBeam is a new single‐source DECT, which may aid in tumor delineation for radiation therapy treatment planning. TwinBeam shows promise for DECT simulations, which aim to capture a dynamic bolus of contrast. This work demonstrates that VMIs reconstructed using the TwinBeam system provide greater CNR between pancreatic tumors and healthy pancreatic parenchyma than virtual single‐energy CT images. For pancreatic tumors which are historically difficult to differentiate, this increase in CNR may increase the ability to accurately segment these tumors for radiation therapy treatment planning, which has the potential to lead to more effective radiation therapy treatment. However, definitive improvements in tumor delineation cannot be stated without further investigation of pancreas GTV segmentation reproducibility and accuracy.

## CONFLICT OF INTEREST

This work was partially funded by a collaboration with Siemens Healthineers.
